# A study of the predictive role of multiple variables for the incidence of acute kidney injury and its outcomes in Indian patients with ST-elevation myocardial infarction and cardiogenic shock

**DOI:** 10.1186/s43044-024-00557-4

**Published:** 2024-09-09

**Authors:** Kewal Kanabar, Yash Paul Sharma, Darshan Krishnappa, Krishna Santosh, Miren Dhudasia

**Affiliations:** 1 Department of Cardiology, U.N. Mehta Institute of Cardiology and Research Centre, Ahmedabad, 380016 India; 2 Department of Cardiology, Postgraduate Institute of Medical Education and Research, Chandigarh, India; 3 Department of Cardiology, Sri Jayadeva Institute of Cardiovascular Sciences and Research, Bangalore, India; 4 Department of Research, Children’s Hospital of Philadelphia, Philadelphia, PA USA

**Keywords:** Cardiogenic shock, ST-elevation myocardial infarction, AKI, Mortality

## Abstract

**Background:**

Acute kidney injury (AKI) occurs frequently in ST-elevation myocardial infarction with cardiogenic shock (CS-STEMI) and is a strong independent prognostic marker for short and intermediate-term outcomes. Owing to the delayed presentation and limited facilities for primary percutaneous coronary intervention in low- and middle-income countries, the incidence, predictors, and outcome of AKI are likely to be different compared to the developed countries. We performed a post hoc analysis of patients presenting with CS-STEMI over 7 years (2016–2022) at a tertiary referral center in North India. The primary outcome assessed was AKI and the secondary outcome was in-hospital mortality.

**Results:**

Of the 426 patients, 194 (45.5%) patients developed AKI, as defined by the Kidney Disease Improving Global Outcomes criteria. Left ventricular (LV) pump failure with pulmonary edema [Odds ratio (OR) 1.67; 95% confidence interval (CI) 1.04–2.67], LV ejection fraction (OR 1.35 per 10% decrease in ejection fraction; CI 1.04–1.73), complete heart block (OR 2.06; CI 1.2–3.53), right ventricular infarction (OR 2.76; CI 1.39–5.49), mechanical complications (OR 3.89; CI 1.85–8.21), ventricular tachycardia (OR 2.80; CI 1.57–4.99), and non-revascularization (OR 2.2; CI 1.33–3.67) were independent predictors of AKI in multivariate logistic regression analysis. Additionally, AKI was a strong predictor of in-hospital mortality (univariate OR 30.61, CI 17.37–53.95).

**Conclusions:**

There is a higher incidence of AKI in CS-STEMI in resource-limited settings and is associated with adverse short-term outcomes. Additional studies are needed to address the optimal strategies for the prevention and management of AKI in such settings.

## Background

Cardiogenic shock (CS) is the leading cause of mortality and adverse outcomes in ST-elevation myocardial infarction (STEMI). Although the outcomes of cardiogenic shock complicating STEMI (CS-STEMI) have improved considerably over the past two decades, they remain sub-optimal [1–4], especially in low- and middle-income countries such as India, which have limited access to primary percutaneous coronary intervention (PCI) and mechanical circulatory support (MCS) devices [5]. Renal function is an important variable and end-point in the context of CS [6]. In contrast with chronic heart failure, the interplay between acute myocardial dysfunction, hypotension, and renal function gets magnified in CS-STEMI. The renal hypoperfusion secondary to low cardiac output, and raised central and renal venous pressures causes acute deterioration of renal function and acute kidney injury (AKI) [7–9]. Other contributory factors may include contrast use and systemic atheroembolism during percutaneous coronary intervention (PCI), cardiopulmonary bypass (CPB) during coronary artery bypass grafting (CABG), and positive pressure ventilation (PPV) [10–12].

Studies have demonstrated that AKI is one of the strongest predictors of both short- and long-term outcomes and the long-term need for chronic dialysis in CS-STEMI [5, 7, 13–18] and acts not only as a surrogate marker of the severity of CS but also as a direct mediator of adverse outcomes. Early (< 24 h) development of AKI after the onset of CS is associated with an especially high likelihood of mortality [19]. Additionally, AKI is also associated with a longer duration of hospital stay and hospitalization costs [15].

Compared to the developed world, patients with STEMI in India present later, and are less likely to receive primary PCI or emergency CABG. Hence, they have a higher likelihood of developing AKI. [5, 20–22] We have previously demonstrated the role of AKI in the prognosis of CS-STEMI in a smaller subgroup of late-presenting patients with CS-STEMI [5, 23]. We aimed to study the predictive role of multiple variables for the incidence of AKI and its outcomes in Indian patients with CS-STEMI.

## Methods

### Study design

We performed a post hoc analysis of a single-center registry of patients with CS-STEMI presenting to a tertiary referral hospital in North India. The study conforms to the ethical guidelines of the Declaration of Helsinki and was reviewed and cleared by the institutional ethics committee of the Postgraduate Institute of Medical Education and Research, Chandigarh, India. Informed written consent was obtained from all patients or appropriate legally authorized representatives.

### Patient selection and evaluation

**Fig. 1:**
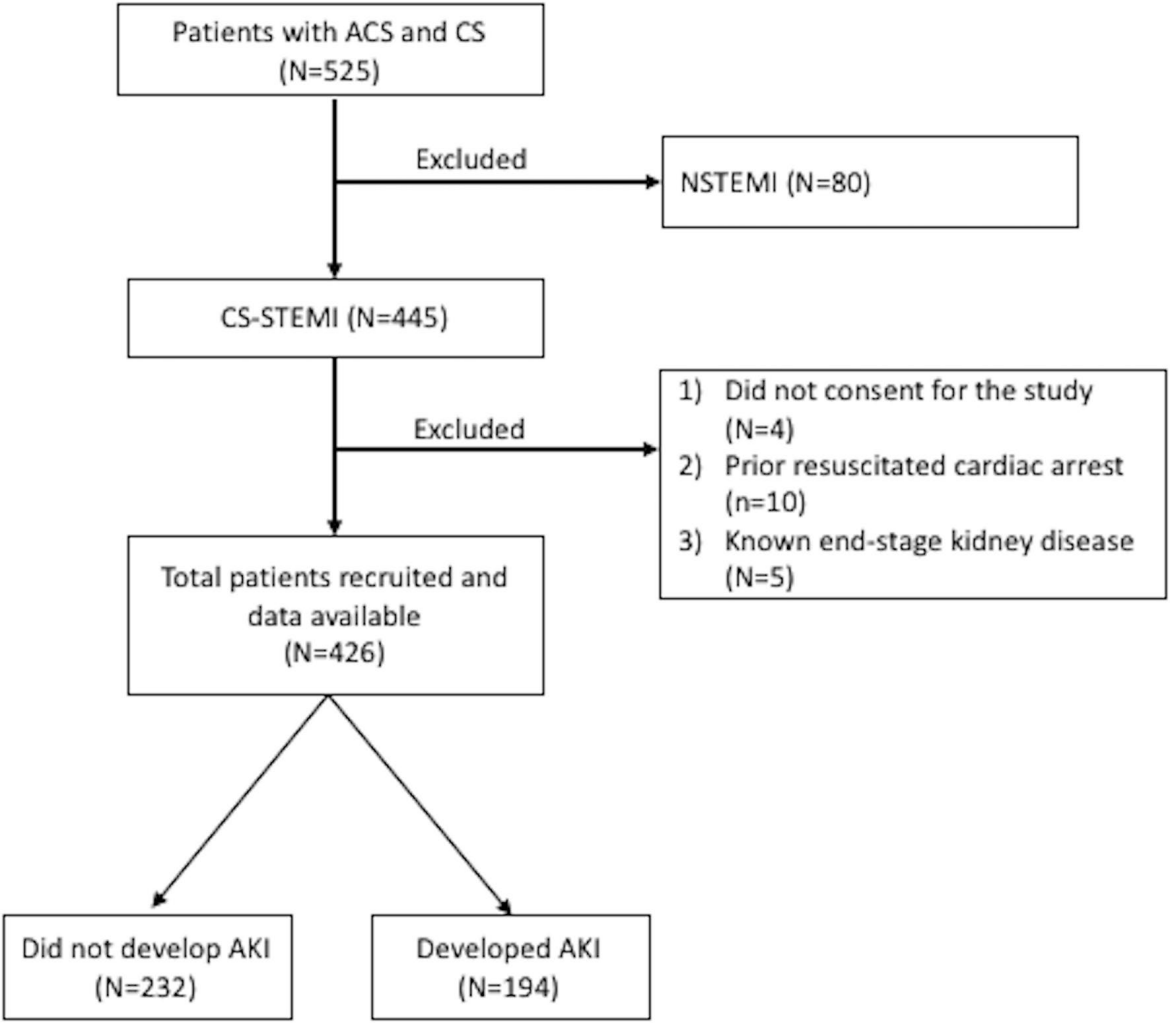
STROBE diagram of the patients in the study. ACS = acute coronary syndrome; CS = cardiogenic shock; NSTEMI = Non-ST-elevation myocardial infarction; CS-STEMI = ST-elevation myocardial infarction complicated by cardiogenic shock

The study enrolled consecutive patients with CS-STEMI over 7 years (January 2016 to December 2022). STEMI was defined by the European Society of Cardiology/ the American College of Cardiology / the American Heart Association/ World Heart Federation (ESC/ACCF/AHA/WHF) third universal definition [24]. We considered CS as systolic blood pressure (SBP) < 90 mm Hg for at least 30 min or the need for supportive measures to maintain SBP > 90 mm Hg despite adequate filling pressures, and signs of end-organ hypoperfusion (oliguria/cold extremities/altered sensorium) [25]. Patients with non-ST-elevation MI (NSTEMI), prior resuscitated cardiac arrest, and known end-stage renal disease (ESRD) were excluded (Fig. 1). Left ventricular (LV) ejection fraction was calculated by the Simpson's method using a 2-D transthoracic echocardiogram (Vivid Q, GE Healthcare, New York, USA). The severity of mitral regurgitation (MR) was graded by the established criteria [26].

Standard tests including hemoglobin, complete blood counts, renal function tests (serum urea and creatinine), liver function tests, high-sensitivity troponin-I, creatine kinase-MB (CK-MB), and coagulation parameters were performed upon hospital admission. Serum urea and creatinine were repeated during the hospital stay every 24–48 h for the initial 3–4 days and then as dictated by the clinical circumstances. The monitoring of urine output was done hourly by the trained nursing staff. The estimated glomerular filtrate rate (eGFR) was calculated using the chronic kidney disease epidemiology collaboration (CKD-EPI) equation [27]. AKI was defined as per the Kidney Disease: Improving Global Outcomes (KDIGO) definition as an increase in serum creatinine by 0.3 mg/dl within 48 h or 1.5 times baseline in last 7 days or urine output £ 0.5 ml/kg/h for 6 h [28].

### Management strategy

The management, including the use of inotropes and circulatory assist devices, and timing of revascularization was decided by the treating physician, based on the delay in presentation, hemodynamic variables, end-organ failure including AKI, neurological status, mechanical complications, and patient willingness [5]. The decision regarding the initiation of renal replacement therapy was taken in consultation with a nephrologist based on acid–base balance, fluid and electrolyte status, urine output, and renal function tests. All patients were treated according to guideline-directed medical treatment and PCI or fibrinolysis for patients not consenting to PCI. The default revascularization strategy was culprit vessel primary PCI. Non-ionic, iso-osmolar (Iodixanol), or low-osmolar (Iohexol) contrast agents were used for performing angiograms and PCI.

Baseline characteristics and in-hospital courses were recorded by trained physicians (cardiology fellows). The angiographic profile was analyzed by trained interventional cardiologists. The primary outcome was AKI; while, the secondary outcome included in-hospital mortality.

### Statistical analyses

Data was entered into a spreadsheet (Microsoft Excel 2016, Microsoft Corporation, USA), and statistical analysis was done using the Statistical Package for Social Sciences (SPSS Inc. version 23.0, IBM Corporation, Chicago, USA). All continuous variables were described as mean (SD) or median [interquartile range (IQ)] and categorical variables as percentages and counts. The difference between two groups for a continuous variable was assessed using the independent t test (parametric) or Mann–Whitney test (non parametric) and between two categorical variables with chi-square or Fisher's exact test. A binary logistic regression analysis was performed to determine the independent predictors for AKI and included variables with *p* ≤ 0.10 on univariate analysis. All other *p*-values are two-tailed and set at a statistical significance of 0.05.

## Results

A total of 426 patients were included, of whom 194 (45.5%) developed AKI (Fig. 1). The cohort was divided into two groups: Group 1 [those who did not develop AKI (*n* = 232)] and Group 2 [those who developed AKI (*n* = 194)].

### Baseline clinical characteristics

The patients in group 2 who developed AKI were older, more likely to be female, diabetic, hypertensive, late presenters, had lower LV ejection fraction, a higher chance of LV pump failure with pulmonary edema, and right ventricular MI compared to group 1. The group 2 had a higher incidence of MI-associated left bundle branch block (LBBB) (5.2% vs. 0.9%; *p* = 0.008), complete heart block (CHB) [28.9% vs. 19.8%; *p* = 0.03], ventricular tachycardia (VT) [26.3% vs. 11.2%; *p* =  < 0.001], and mechanical complications [severe MR (10.2% vs. 3%; *p* = 0.002) and ventricular septal rupture (5.2% vs. 1.7%; *p* = 0.048)]. The differences in the location of MI (57.8% vs. 60.3; *p* = 0.59) and the proportion of patients who received fibrinolysis (50.4% vs. 45.9%; *p* = 0.34) between the two groups were statistically insignificant (Table 1).

**Table 1:** Comparison between patients with and without AKI

Characteristic	Group 1 (No AKI) *N* = 232	Group 2 (AKI) *N* = 194	*P*-value
Age, years, mean (SD)	58.33 (12.18)	60.63 (11.38)	0.046
Sex, *n* (%)			
Male	181 (78)	125 (65.4)	0.002
Female	51 (22)	69 (35.6)	
Risk factors, *n* (%)			
Diabetes Mellitus	77 (33.2)	88 (45.4)	0.01
Hypertension	89 (38.4)	98 (50.5)	0.01
Smoking	113 (48.7)	73 (37.6)	0.02
Family History of CAD	19 (8.2)	15 (7.7)	0.86
Prior CVA	5 (2.2)	7 (3.6)	0.36
Prior MI	20 (8.6)	17 (8.8)	0.95
Prior PCI/CABG	5 (2.2)	7 (3.6)	0.36
Time to presentation, hours, median (IQ)	12 (6–24)	15 (6–36)	0.07
Anterior MI, *n* (%)	134 (57.8)	117 (60.3)	0.59
LV pump failure with pulmonary edema, *n* (%)	109 (47)	129 (66.5)	< 0.001
LV ejection fraction, %, mean ± SD	32.65 ± 10.2	29.8 ± 10.03	0.004
Fibrinolysis, *n* (%)	117 (50.4)	89 (45.9)	0.34
Conduction abnormalities, *n* (%)			
RBBB	25 (10.9)	31 (16)	0.11
LBBB	2 (0.9)	10 (5.2)	0.008
CHB	46 (19.8)	56 (28.9)	0.03
Right ventricular MI, *n* (%)	22 (9.5)	33 (17)	0.02
Ventricular tachycardia, *n* (%)	26 (11.2)	51 (26.3)	< 0.001
Mechanical complications, *n* (%)	13 (5.6)	35 (18)	< 0.001
Severe MR	7 (3)	20 (10.2)	0.002
Ventricular septal rupture	4 (1.7)	10 (5.2)	0.048
Cardiac rupture	2 (0.9)	5 (2.6)	0.16
Coronary angiogram, *n* (%)	182 (78.4)	81 (41.8)	< 0.001
Creatinine, mg/dl, mean ± SD	1.1 ± 0.61	2.0 ± 1.29	< 0.001
eGFR, mL/min/1.73m^2^, median (IQ)	76 (52.3–99.3)	39.5 (25.9–58.1)	< 0.001

*AKI* Acute kidney injury, *CABG* Coronary artery bypass grafting, *CAD* Coronary artery disease, *CHB* Complete heart block, *CVA* Cerebrovascular accident, *eGFR* estimated glomerular filtrate rate, *IQ* Interquartile range, *LBBB* Left bundle branch block, *LV* Left ventricular, *MI* Myocardial infarction, *MR* Mitral regurgitation, *PCI* Percutaneous coronary intervention, *RBBB* Right bundle branch block

### Angiographic profile

Patients in group 2 were less likely to undergo an angiogram (41.8% vs. 78.4%; *p* = 0.001) and PCI (24.7% vs. 48.7%; *p* = 0.001) and more likely to have an intra-aortic balloon pump (IABP) implanted. The IABP was the only assist device used because of the non-availability of other support devices. The culprit vessel and pattern of coronary involvement were not significantly different between the two groups (Table 2).

**Table 2:** Comparison of angiographic characteristics between patients with and without AKI

Characteristic	No AKI *N* = 232	AKI *N* = 194	*P*-value
Coronary angiogram, *n* (%)	182 (78.4)	81 (41.8)	< 0.001
Culprit lesion, *n* (%)*			
LAD	100 (54.9)	42 (51.9)	0.80
LCX	12 (6.6)	7 (8.6)	
RCA	70 (38.5)	32 (39.5)	
Coronary artery involved, *n* (%)*			
LMCA	6 (3.3)	7 (8.6)	0.11
LAD	136 (74.7)	60 (74.1)	0.91
LCX	64 (35.2)	37 (45.7)	0.10
RCA	115 (63.2)	52(64.2)	0.87
Type of vessel involvement, *n* (%)*			
Single-vessel disease	88 (48.4)	32 (39.5)	0.18
Double-vessel disease	55 (30.2)	29 (35.8)	0.37
Triple-vessel disease	39 (21.5)	20 (24.7)	0.60
LMCA/Multi-vessel disease	95 (52.2)	52 (64.2)	0.07
Occlusion of culprit vessel, *n* (%)*	66 (36.5)	36 (44.4)	0.22
CTO of non-culprit vessel, *n* (%)*	15 (8.2)	13 (16)	0.058
PCI, *n* (%)	113 (48.7)	48 (24.7)	< 0.001
GP IIb/IIIa inhibitor use, *n* (%)	72 (31)	48 (24.7)	0.15
IABP use, *n* (%)	14 (6)	29 (14.9)	0.002

*AKI* Acute kidney injury, *CTO* Chronic total occlusion, *IABP* Intra-aortic balloon pump, *LAD* Left anterior descending artery, *LCX* Left circumflex artery, *LMCA* Left main coronary artery, *PCI* Percutaneous coronary intervention, *RCA* Right coronary artery

^*^Values are based on 263 patients (182 without and 81 with AKI) who underwent an angiogram. More than 70% stenosis of the LAD, RCA, LCX, and more than 50% stenosis of the LMCA was considered significant

### Multivariate analysis

**Fig. 2:**
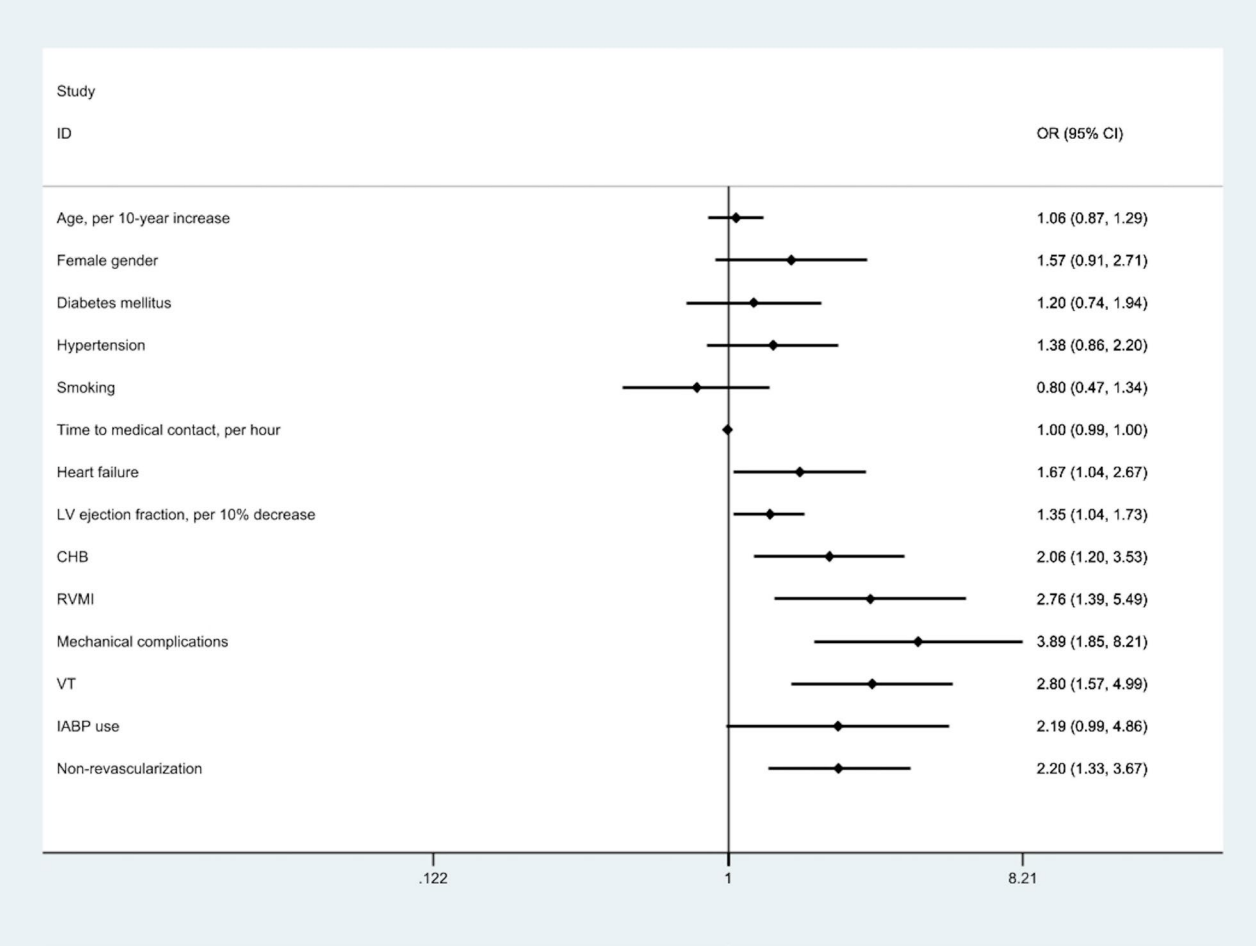
Predictors of acute kidney injury in STEMI with cardiogenic shock. CHB = complete heart block; CI = confidence interval; IABP = intra-aortic balloon pump; LV = left ventricular; OR = odds ratio; RVMI = right ventricular myocardial infarction; VT = ventricular tachycardia

A binary logistic regression analysis was performed to determine the effects of age, gender, diabetes, hypertension, smoking, time to presentation, LV ejection fraction, LV failure, LBBB, CHB, RVMI, mechanical complications, VT, IABP use, left main/multivessel disease and non-revascularization on AKI. Of these, left ventricular (LV) pump failure with pulmonary edema [Odds ratio (OR) 1.67; 95% confidence interval (CI) 1.04–2.67], LV ejection fraction (OR 1.35 per 10% decrease in ejection fraction; CI 1.04–1.73), CHB (OR 2.06; CI 1.2–3.53), RVMI (OR 2.76; CI 1.39–5.49), mechanical complications (OR 3.89; CI 1.85–8.21), VT (OR 2.80; CI 1.57–4.99), and non-revascularization (OR 2.2; CI 1.33–3.67) were independent predictors of AKI in multivariate regression analysis (Table 3 and Fig. 2). The overall in-hospital mortality was 38% and was significantly higher in patients with AKI compared to those without (73.2% vs. 8.2%; *p* < 0.001; univariate OR of AKI for in-hospital mortality 30.61, CI 17.37–53.95).

**Table 3:** Logistic regression analysis for independent predictors of AKI

Variable	Odds Ratios	C.I	*P*-value
Mechanical complications	3.89	1.85–8.21	< *0.001*
Ventricular tachycardia	2.80	1.57–4.99	< *0.001*
Right ventricular MI	2.76	1.39–5.49	*0.004*
Non-revascularization	2.2	1.33–3.67	*0.002*
Complete heart block	2.06	1.2–3.53	*0.009*
Left ventricular (LV) pump failure with pulmonary edema	1.67	1.04–2.67	*0.03*
LV ejection fraction, per 10% decrease	1.35	1.04–1.73	*0.027*
Age, per 10-year increase	1.06	0.87–1.29	0.55
Female gender	1.57	0.91–2.71	0.10
Diabetes mellitus	1.20	0.74–1.94	0.45
Hypertension	1.38	0.86–2.20	0.179
Smoking	0.80	0.47–1.34	0.40
Time to medical contact, per hour	0.997	0.99–1.003	0.32
LBBB	4.74	0.79–28.49	0.09
IABP use	2.19	0.99–4.86	0.052
LMCA/multi-vessel disease	1.56	0.8–3.01	0.184

Italic value indicates *p* < 0.05

*AKI* Acute kidney injury, *CI* Confidence interval, *IABP* Intra-aortic balloon pump, *LBBB* Left bundle branch block, *LMCA* Left main coronary artery, *LV* Left ventricular, *MI* Myocardial infarction

The linearity of the continuous variables with respect to the logit of the dependent variable was analyzed using the Box–Tidwell procedure. A Bonferroni correction was applied using all 20 elements in the model resulting in statistical significance being accepted when *p* < .0025. Based on this assessment, all continuous independent variables were found to be linearly related to the logit of the dependent variable. The logistic regression model was statistically significant [χ^2^(4) = 28.36, *p* < .0001]. The model explained 52% (Nagelkerke *R*^*2*^) of the variance in in-hospital mortality and correctly classified 87.5% of cases

## Discussion

In a 7-year study of CS-STEMI at a tertiary referral hospital, we found that 45% of an unselected cohort of patients either presented with/developed AKI during the hospital stay and had significantly higher in-hospital mortality compared to those without AKI. Left ventricular failure, LV ejection fraction, CHB, RVMI, mechanical complications, VT, and non-revascularization were associated with a higher risk of AKI.

“The incidence rate of AKI in our study was significantly higher than that reported from studies performed in the developed countries [15, 17, 22]. This may be attributed to a significant delay in presentation and lower rates of revascularization. Also, the predominance of fibrinolysis as a revascularization strategy may have resulted in a larger number of patients with unsuccessful revascularization persistent occlusion of the culprit vessel, and higher chances of AKI. Previous studies have demonstrated that single- or multi-organ failure, especially renal failure is associated with higher in-hospital mortality and resource utilization in CS-STEMI [5, 7, 15, 18, 19, 29]. Also, AKI is associated with significant post-discharge resource utilization and 30-day readmission [15, 17, 30]. Our findings of significantly higher in-hospital mortality in patients with AKI are in line with the established literature (Table 4). The lower rates of coronary angiogram and PCI in patients with AKI reflect the reluctance to perform an angiogram due to the higher anticipated risk [15].

**Table 4:** Overview of studies assessing predictors and outcomes of AKI in CS-STEMI

Study	No. of patients	AKI incidence (%)	Predictors of AKI	Mortality (AKI vs. no AKI)
Hochman et al. [22]	302	18	–	–
Koreny et al. [19]	118	33	Age > 75, diabetes, mechanical complications, mechanical ventilation	87% versus 53% AKI only independent predictor of mortality
Marenzi et al. [18]	97	55	Age > 75, LV ejection fraction < 40%, mechanical ventilation	50% versus 2.2% AKI strongest predictor of mortality
Lauridosn et al. [17]	5079	13	–	62 versus 36% 5-year risk of dialysis 11% versus 1%
Arbel et al. [34]	48	60	Positive fluid balance	Positive fluid balance associated with higher mortality (68% vs. 10%) and low likelihood of recovery of renal function
Margolis et al. [35]	84	60	Positive fluid balance	Positive fluid balance associated with low likelihood of recovery of renal function
Tarvasmaki et al. [9]	125	31	Age, CKD, lower arterial pH, higher CVP	AKI associated with high mortality
Van den Akker et al. [8]	39	61.5	CVP	38% versus 7%
Vallabhajosyula et al. [15]	440257	35	Age, black race, non-private insurance, higher comorbidity, organ failure, cardiac and non-cardiac organ support	46% versus 34%
Adegbala et al. [7]	8900	6	–	76% versus 51%
Current study	426	45	LV failure with pulmonary edema, LV ejection fraction, CHB, RVMI, mechanical complications, VT, non-revascularization	73% vs 8%

*AKI* Acute kidney injury, *CHB* Complete heart block, *CKD* Chronic kidney disease, *CS-STEMI* Cardiogenic shock complicating ST-elevation myocardial infarction, *CVP* Central venous pressure, *LV* Left ventricular, *MI* Myocardial infraction, *RVMI* Right ventricular MI, *VT* Ventricular tachycardia

Table 4 shows a comparison of the current with the results of our study with the results of a number of previous relevant studies. Although higher age, male sex, and comorbidities are associated with AKI [9, 15, 19, 31], our study may not have been adequately powered to reveal these differences. The slightly higher rates of AKI in females may have been due to lower rates of revascularization compared to males. Heart failure, LV ejection fraction, and ventricular arrhythmias have previously been shown to be independent correlates of AKI [18, 19, 32]. Complete heart block, mechanical complications, and RVMI cause CS due to low cardiac output and predispose to the development of AKI. High right atrial pressure in RVMI has been shown to lead to worsening renal functions in CS-STEMI [33]. Although central venous pressure and mechanical ventilation are other strong predictors of AKI, we did not assess these in our study. Since baseline serum creatinine and eGFR were not available for many patients, we did not assess the prognostic role of chronic kidney disease (CKD) in the study.

We assessed the renal function and eGFR using serum creatinine measurement only. Although several novel biomarkers such as neutrophil gelatinase-associated lipocalin (NGAL), kidney injury molecule-1 (KIM-1), and cystatin C help in the assessment of renal function, serum creatinine is the most widely utilized marker and is the strongest predictor of 30-day and 12-month mortality and need of renal replacement therapy at 30 days [6, 13, 16]. Hence, the newer biomarkers do not provide any incremental prognostic value over the measurement of serum creatinine.

There are several limitations to the study. This was a post hoc analysis of single-center data with a possible referral bias since it caters to a large population and most of the patients are referred from smaller centers. The low rates of revascularization due to late presentation, lack of adequate health insurance, and non-availability of assistive devices other than IABP may have affected the findings of the study. Also, we did not distinguish between AKI due to CS or due to the use of contrast agents during cardiac catheterization. Also, since we did not record the contrast volume, its impact on renal function remains unknown. A detailed assessment of hemodynamics may have yielded additional prognostic variables. Additionally, we did not assess other biomarkers such as NGAL, KIM-1, and cystatin C due to logistic issues. The impact of Extracorporeal membrane oxygenation (ECMO) and mechanical circulatory support on the incidence and outcomes of AKI was not assessed, as done by several authors [9, 36–38]. Also, the impact of renal replacement therapy on intermediate-term outcomes remains unknown in the current study since we only assessed the in-hospital outcomes. Since follow-up details were not recorded, intermediate- and long-term mortality and the need for renal replacement therapy are unknown.

## Conclusions

We found a high incidence of AKI in patients presenting at a tertiary center with CS-STEMI in a low-middle-income country. Acute kidney injury was a strong predictor of in-hospital mortality. Heart failure, LV ejection fraction, CHB, RVMI, mechanical complications, VT, and non-revascularization were independent predictors of the development of AKI. As AKI is likely to be a common problem in CS-STEMI in resource-limited settings, establishing the ideal prevention and management strategies with appropriate studies remains pertinent.

## Data Availability

The datasets used and/or analyzed during the current study are available from the corresponding author on reasonable request.

## Abbreviations

AKI: Acute kidney injury
CS-STEMI: ST-elevation myocardial infarction with cardiogenic shock
LV: Left ventricular
KDIGO: Kidney Disease Improving Global Outcomes
OR: Odds ratio
PCI: Primary percutaneous coronary intervention
MCS: Mechanical circulatory support
CPB: Cardiopulmonary bypass
PPV: Positive pressure ventilation
CABG: Coronary artery bypass grafting
MR: Mitral regurgitation
ESC/ACCF/AHA/WHF: European Society of Cardiology/ the American College of Cardiology / the American Heart Association/ World Heart Federation
SBP: Systolic blood pressure
ESRD: End-stage renal disease
CK-MB: Creatine kinase–myocardial band
eGFR: Estimated glomerular filtrate rate
CKD-EPI: Chronic kidney disease epidemiology collaboration
LBBB: Left bundle branch block
CHB: Complete heart block
VT: Ventricular tachycardia
IABP: Intra-aortic balloon pump
RVMI: Right ventricular myocardial infarction
CKD: Chronic kidney disease
NGAL: Neutrophil gelatinase-associated lipocalin
KIM-1: Kidney injury molecule-1
ECMO: Extracorporeal membrane oxygenation
